# Pir2/Rnf144b is a potential endometrial cancer biomarker that promotes cell proliferation

**DOI:** 10.1038/s41419-018-0521-1

**Published:** 2018-05-02

**Authors:** Qing Zhou, Sahar Eldakhakhny, Franco Conforti, Emma J. Crosbie, Gerry Melino, Berna S. Sayan

**Affiliations:** 1 University of Manchester, Faculty of Medical and Human Sciences, Division of Cancer Sciences, Manchester Cancer Research Centre, Wilmslow Road, Manchester, M20 4QL UK; 2 University of Southampton, Faculty of Medicine, Academic Unit of Clinical and Experimental Sciences, Southampton, SO16 6YD UK; 3 Medical Research Council, Toxicology Unit, Hodgkin Building, Leicester University, Lancaster Road, P.O. Box 138, Leicester, LE1 9HN UK; 4 Biochemistry Laboratory, IDI-IRCCS, and Department of Experimental Medicine and Biochemical Sciences, University of Rome “Tor Vergata”, 00133 Rome, Italy

## Abstract

Endometrial cancer is one of the most common gynaecological cancers in developed countries. Its incidence has increased 20% over the last decade and the death rate has increased >100% over the past two decades. Current models for prediction of prognosis and treatment response are suboptimal, and as such biomarkers to support clinical decision-making and contribute to individualised treatment are needed. In this study, we show that the E3-ubiquitin ligase PIR2/RNF144B is a potential targetable biomarker in endometrial cancer. At transcript level, it is expressed both in normal endometrium and tumour samples, but at protein level, it is expressed in tumours only. By using endometrial cancer cell lines, we demonstrated that PIR2/RNF144B is stabilised via phosphorylation downstream of GSK3β and this is necessary for the proliferation of endometrial cancer cells, in the absence of oestrogenic growth stimuli. Here, inactivation of GSK3β activity is associated with loss of PIR2/RNF144B protein and consequent inhibition of cell proliferation. Our results, therefore, substantiate PIR2/RNF144B as a novel candidate for targeted therapy in endometrial cancer.

## Introduction

Endometrial cancer (EC) is one of the most common gynaecological cancers worldwide and its incidence has risen by more than 50% over the last 2 decades^1,2^. Although most women diagnosed with EC present with early-stage disease confined to the uterus, metastatic disease is identified in around 25% when comprehensive staging is performed. The 5-year overall survival for these women is extremely poor at around 20–26%^3,4^. Current treatment for advanced EC is limited to surgery followed by chemotherapy and radiotherapy, with very few novel targeted therapies under evaluation. A better understanding of EC is urgently needed to develop novel, efficient and effective treatment regimens, particularly for those that have spread or recurred.

EC is broadly divided into 2 types based on clinico-pathological and molecular characteristics^5,6^. Type I ECs, which account for ~80% of all cases, are driven by excessive stimulation of the endometrium by oestrogens synthesised in the fat tissue of obese women^7–9^. Type II ECs, on the other hand, are frequently associated with p53 and p16 mutations and are oestrogen/oestrogen receptor (ER)-independent^10,11^.

ER status in Type I EC is an important prognostic factor and higher level of ERα predicts favourable survival^12–14^. While low-grade Type I tumours are strongly positive for ER, its expression is lost in higher-grade tumours^15,16^. Phosphatase and tensin homolog (PTEN) mutations are also common in Type I ECs, >80% of tumours harbouring mutations targeting this pathway^5,17^. PTEN functions as a lipid and protein phosphatase, inhibiting the ability of PDK1 to activate AKT. Loss of PTEN function results in constitutive AKT activation and phosphorylation of downstream targets, and hence promoting proliferation^18–20^.

The serine/threonine kinase GSK3β is amongst the targets of AKT. In normal uterine epithelial cells, AKT-GSK3β signalling pathway is regulated by the actions of oestrogen and progesterone to regulate the sub-cellular localisation of cyclin D1, and hence proliferation^21^. Here, activation of AKT downstream of ER inhibits GSK3β, which is essential for cell cycle progression^21^. As such, inhibition of GSK3β activity induces uterine epithelial cell proliferation in human endometrial tissue xenografts^22^ and in parallel to this observation, it has been reported that women who had been treated with mood stabilizers, such as the GSK3β inhibitor lithium chloride, display higher incidence of endometrial hyperplasia^23^. Conversely, in EC, inhibition of GSK3β activity is associated with inhibition of cell proliferation both in vitro^24^ and in vivo^25^ and GSK3β has been shown to be overexpressed in EC, which is positively related to the stage of cancer and negatively related to relapse-free survival rate^25,26^. These interesting observations warrant further research to understand the contradictory function of GSK3β in endometrial tissue.

PIR2/RNF144B (hereafter referred as to PIR2) is an E3-ubiquitin ligase that is important for the regulation of apoptosis and cell proliferation^27–29^. It is highly expressed at the basal layer of the epidermis and in head and neck squamous carcinoma (HNSCC) cells, where it regulates proliferation and differentiation^29^. Its oncogenic role has also recently been shown in chordoma, where its depletion results in impaired cell proliferation^30^. Here we show that PIR2 protein is not expressed in normal endometrium, but expressed only in EC. In EC cell lines, PIR2 drives cell proliferation when oestrogen-mediated growth signalling is lost. By in silico analysis, mass spectrometry and kinase library screening, we identified that PIR2 is phosphorylated downstream of GSK3β and phosphorylated PIR2 is protected from proteasomal degradation, leading to its accumulation. Our findings suggest that PIR2 can potentially be used as a biomarker for endometrial cancer and inhibition of its expression may offer novel therapeutic approaches for the treatment of the disease.

## Results

### PIR2 is a potential endometrial cancer biomarker that drives proliferation

**Fig. 1:**
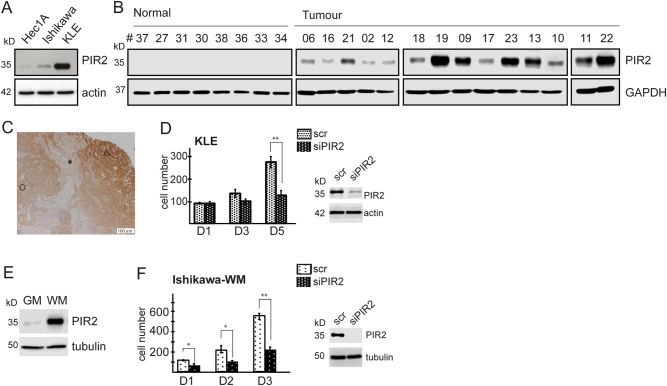
PIR2 is a potential EC biomarker that promotes proliferation. **a** Western blot analysis of PIR2 in a panel of EC cell lines. Actin was used as equal loading control. **b** Equal amount of protein from normal and malignant endometrial tissue was used to analyse PIR2 protein levels by western blotting. **c** IHC showing that PIR2 is mainly localised to the cytoplasm in tumour cells. Necrotic area (Δ), tumour stroma (*), non-necrosis area (O). The scale bar represents 100 um. **d** KLE cells were transfected with scrambled control (scr) or siPIR2. 24 h later cells were plated as triplicates into 24-well plates and fixed at indicated time points. DAPI-stained cells were then counted and the numbers were plotted on graphs, equalising the number of initially plated cells to 100. PIR2-depletion efficiency was assessed by comparing PIR2 protein levels on the final day of the experiment. **e** Ishikawa cells growing in growth media (GM) or in withdrawal media (WM) were lysed and PIR2 expression was assessed by western blotting. **f** Proliferation assay showing the growth dynamics of Ishikawa cells in GM or WM, following transfection with scrambled control or siPIR2. Cells, plated in triplicates, were fixed on indicated days and counted following DAPI staining. Error bars represent standard deviations of experiment replicates (*n* = 3). Western blot shows PIR2 levels on the third day of the experiment. (*Pvalue < 0.05, **P value < 0.01, calculated by Student’s *t*test. Error bars represent standard deviations of experiment replicates)

On the basis of the role of PIR2 in the regulation of proliferation and apoptosis, we investigated its expression profile in a panel of cell lines and identified that it is expressed in endometrial cancer (EC) cell lines, albeit at various levels (Fig. 1a). This led us to investigate PIR2 expression pattern in primary tissues. Analysis of normal (*n* = 8) and malignant (*n* = 14) endometrial tissue samples revealed that PIR2 protein is present exclusively in tumour samples but not in normal endometrium (Fig. 1b). In EC tumour samples, PIR2 is predominantly localised to cytoplasm, as described previously^29^ (Fig. 1c). These data suggested that PIR2 is a potential EC biomarker and prompted us to investigate the functional significance of PIR2 expression in EC.

To assess whether PIR2 expression confers a selective growth or survival advantage to cancer cells, we silenced its expression in KLE cells, which express high levels of PIR2 protein and performed proliferation assays. Loss of PIR2 had a cytostatic effect, which was associated with increased G1 population (Fig. 1d and Supplementary Figure S1A). PIR2-depletion in Hec1A and Ishikawa cells, which express low levels of PIR2 did not have a detectable effect on proliferation (Supplementary Figure S1B). Unlike KLE cells, both Hec1A and Ishikawa cells are ER positive and oestrogen acts as the principal driver for proliferation^31,32^. As such, we hypothesised that the function of PIR2 might become evident in the absence of oestrogenic stimuli. To this end, we starved Ishikawa cells for oestrogen in phenol-red free medium supplemented with charcoal-stripped FBS (withdrawal medium: WM), for 3 days. Surprisingly, lack of oestrogen signalling did not have an impact on Ishikawa cell proliferation, but led to a striking increase in PIR2 levels (Supplementary Figure S1C and Fig. 1e), suggesting that upregulation of PIR2 may function to sustain proliferation in the absence of oestrogen. To test this, we inhibited PIR2 upregulation -in response to oestrogen withdrawal- by RNA interference and assessed cell proliferation (Supplementary Figure S1D). While scrambled transfected oestrogen-starved cells that express high levels of PIR2 proliferated efficiently in the absence of hormone-stimuli, cells that were deprived of PIR2 failed to do so (Fig. 1f). These results demonstrate that in the absence of oestrogenic growth stimuli, PIR2 promotes EC cell proliferation and suggest that inhibition of its expression may potentially have therapeutic implications.

### PIR2 expression is regulated predominantly at post-translational level in tumour cells

**Fig. 2:**
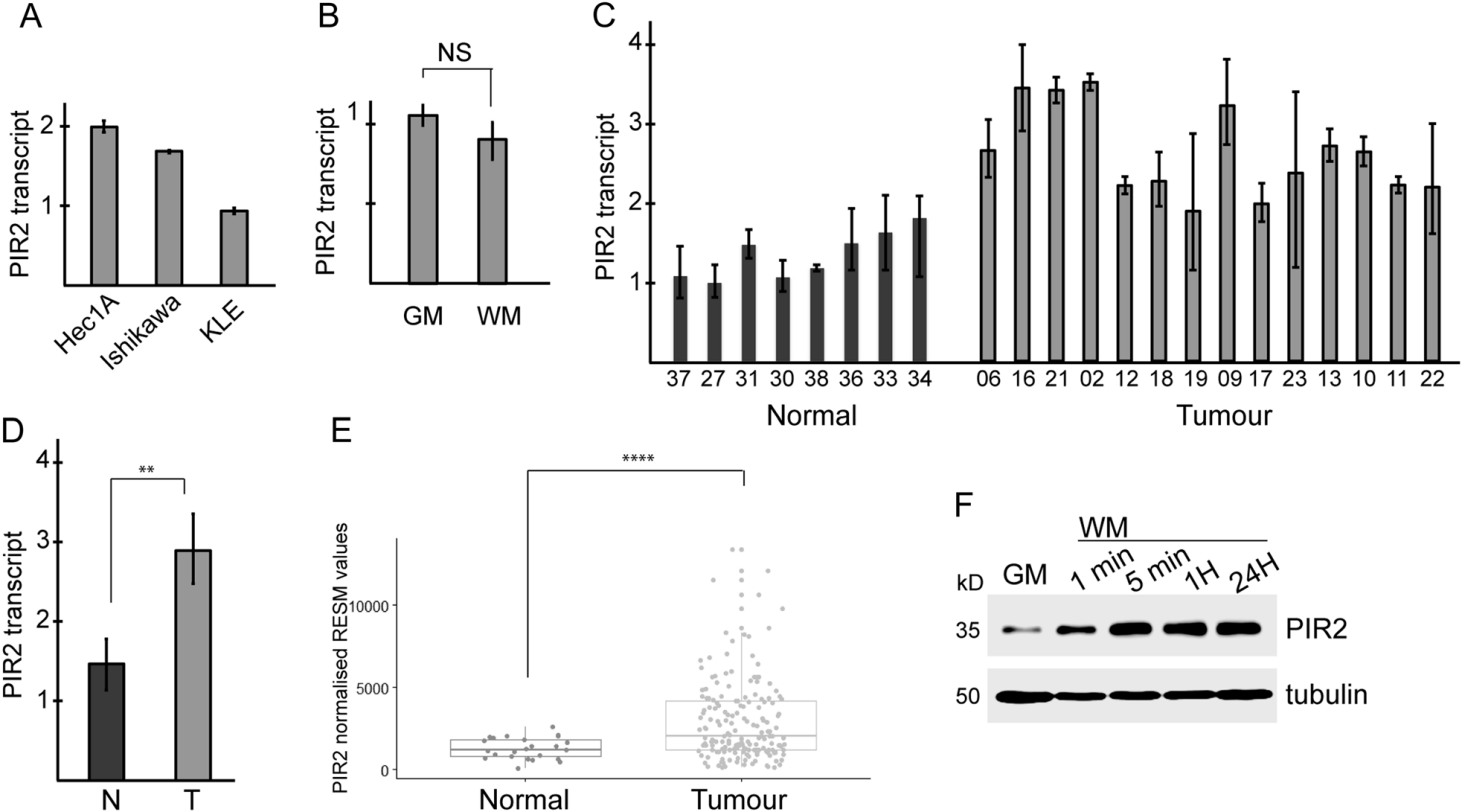
PIR2 transcript levels in EC. **a** qRT-PCR analysis of PIR2 transcript levels in EC cell lines showing relatively lower expression in KLE cells. **b** RNA extracted from Ishikawa cells in growth media (GM) and from Ishikawa cells in withdrawal media (WM) was used to determine changes at PIR2 transcript levels by qRT-PCR. **c**, **d** Expression of PIR2 at RNA level in normal and tumour samples, showing PIR2 is expressed both in normal and tumour samples, although its expression is relatively higher in tumours. **e** Analysis of RNA-seq data from the TCGA-Uterine Corpus Endometrial Carcinoma set. RNA level of PIR2 was found to be increased in the endometrial carcinoma tissue cohort (*n* = 176) compared to the normal tissue cohort (*n* = 24) (*P* = 0.00066). **f** Ishikawa cells were transferred from GM to WM. Cells were collected after 1 min, 5 min, 1 H and 24 H, and cell lysates were analysed for PIR2 expression. Tubulin was used as equal loading control

To investigate how PIR2 can potentially be targeted, we sought to understand the molecular mechanisms that regulate PIR2 expression in EC cells. Here we first assessed potential contribution of transcriptional regulation, by analysing PIR2 transcript levels in EC cell lines, normal endometrium and EC tumours. Although PIR2 protein was hardly detectable in Hec1A and Ishikawa cells, at transcript level, these cell lines expressed relatively higher levels of PIR2 compared to KLE cells, suggesting that, in EC cell lines, PIR2 expression is not regulated transcriptionally (Fig. 2a, Supplementary Figure S2A). Furthermore, starvation of Ishikawa cells for oestrogen did not alter PIR2 mRNA level, although its protein level was increased significantly (Fig. 1e, Fig. 2b, Supplementary Figure S2B). In normal endometrium, PIR2 transcript was readily detectable, albeit this was lower than tumour samples (Fig. 2c, d, Supplementary Figure S2C-D). Analysis of the TCGA-Uterine Corpus Endometrial Carcinoma RNA-seq data provided further support to our findings, showing that PIR2 is present at mRNA level in normal endometrium, although at lower quantities compared to tumour samples (Fig. 2e). These data suggest that both transcriptional and post-translational mechanisms conspire to the upregulation of PIR2 expression during malignant transformation, but in tumour cells post-translational regulation plays a major role.

Modulation of PIR2 protein levels in response to oestrogen withdrawal in Ishikawa cells suggested that we could use this cell line as a model to investigate how PIR2 expression is regulated. To better understand the mode of PIR2 regulation in these cells, we assessed PIR2 protein levels 1-, 5- 60-min after oestrogen withdrawal and compared it to PIR2 levels in cells in GM or in WM for 24 h (Fig. 2f). PIR2 protein levels started to increase within minutes following oestrogen withdrawal, suggesting potential involvement of a kinase-signalling pathway regulating PIR2 protein abundance in EC cells.

### PIR2 is phosphorylated downstream of GSK3β

**Fig. 3:**
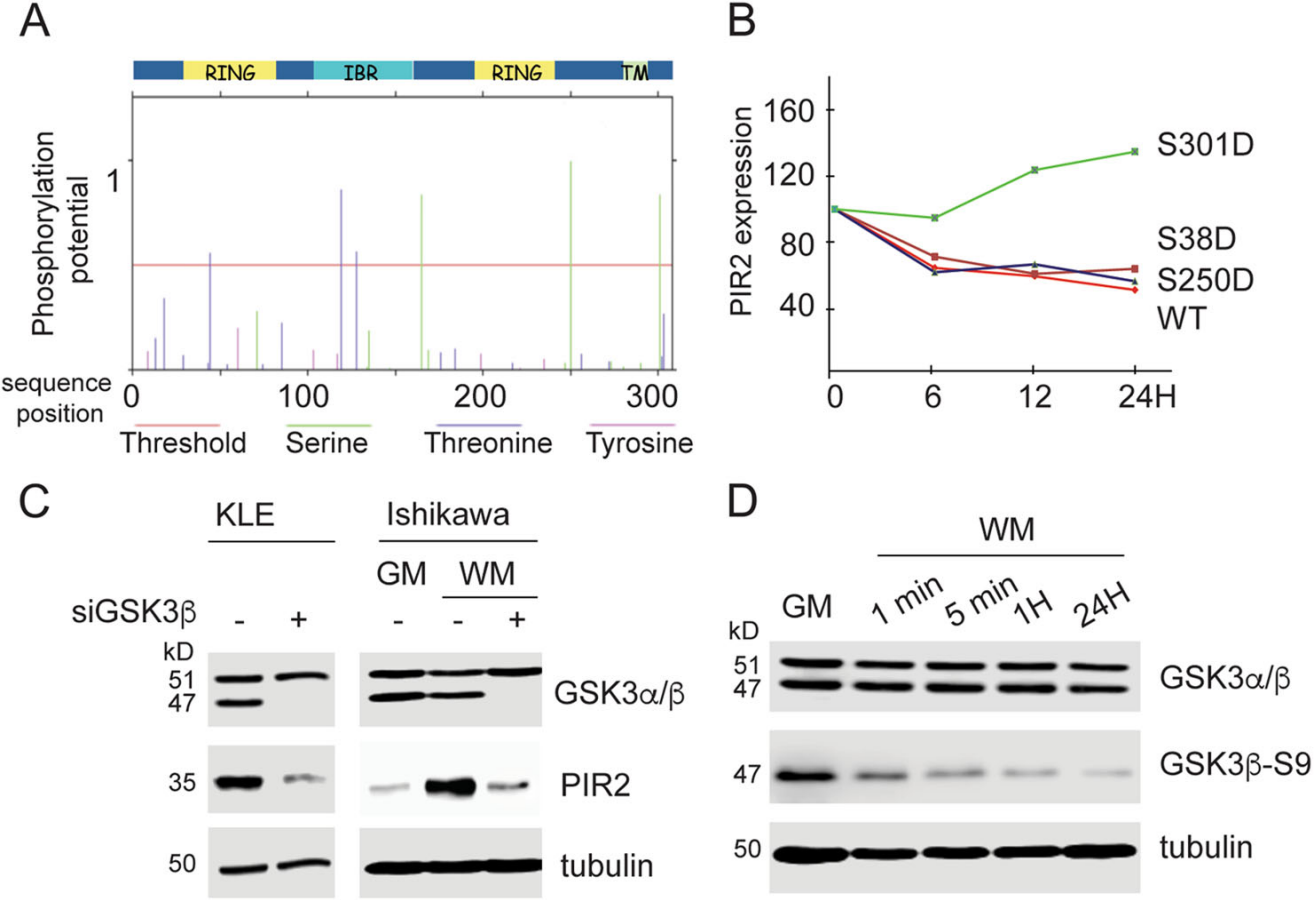
PIR2 is a phosphoprotein. **a** Potential PIR2 phosphorylation sites identified by the NetPhos software. Bars crossing the threshold line represent potential phosphorylation sites. **b** S38, S250 and S301 residues were changed into aspartate residues by site-directed mutagenesis (S38D, S250D and S301D). Wild-type PIR2 and phosphomimetic mutants were overexpressed in H1299 cells. Cells were treated with cycloheximide and collected at 6 H, 12 H and 24 H time points and lysed to assess PIR2 expression by western blotting. The amount of PIR2 was quantitated by using ImageJ, equalised to actin and plotted on the graph. **c** Western blot showing changes in PIR2 protein levels following depletion of GSK3β expression in KLE cells and oestrogen-depleted Ishikawa cells. Cells were transfected with scrambled control siRNA or siRNA targeting GSK3β. In Ishikawa cells, a fraction of cells were taken into WM 48 h after transfection and collected 24 h after oestrogen withdrawal. Lysate from Ishikawa cells in GM was loaded for comparison of PIR2 levels in GM versus GSK3β depleted WM conditions. **d**  Blots  used in Fig. 2f  were re-probed to assess total GSK3β and GSK3β-S9 levels

To assess whether PIR2 expression is regulated downstream of a kinase signalling pathway, we first performed an in silico analysis to investigate the presence of potential phosphorylation sites. By using NetPhos 2.0 (http://www.cbs.dtu.dk/ services/NetPhos/), we identified 3 potential serine phosphorylation sites (S165, S250 and S301) and 3 potential threonine phosphorylation sites (T44, T119 and T128) (Fig. 3a). To validate these findings, we immunoprecipitated overexpressed-PIR2 from HEK293 cells and analysed the immune complexes by mass spectrometry (MS). MS analysis revealed that PIR2 is phosphorylated at three serine residues: S38, S250 and S301. We then tested the impact of phosphorylation at these serine residues on PIR2 protein stability by generating phospho-mimetic PIR2 mutants and comparing the half-life of these mutants to wild-type PIR2. As shown in Fig. 3b and Supplementary Figure S3, only the phospho-mimetic mutation at S301 enhanced the stability of PIR2, while the S38D and S250D mutants had little, if any, effect on the stability of the protein. These findings demonstrate that phosphorylation of PIR2 at S301 is critical for its stability and prompted us to identify the kinase that is responsible for PIR2 phosphorylation and hence, stability.

By screening the PKIS (Published Kinase Inhibitory Set) from GlaxoSmithKline we identified that the inhibition of GSK3β leads to a significant loss of PIR2 protein, suggesting that GSK3β acts as a regulator of PIR2 stability (Supplementary table S1). Depletion of GSK3β in KLE cells resulted in loss of PIR2 protein and depletion of GSK3β in Ishikawa cells inhibited the accumulation of PIR2 protein in response to oestrogen withdrawal (Fig. 3c). In oestrogen-starved cells, accumulation of PIR2 protein paralleled loss of S9-phosphorylated inactive isoform of GSK3β (Fig. 2e and Fig. 3d). These data demonstrate that PIR2 protein stability is associated, predominantly, with the modulation of GSK3β activity.

### Phosphorylation of PIR2 protects it from proteasomal degradation

**Fig. 4:**
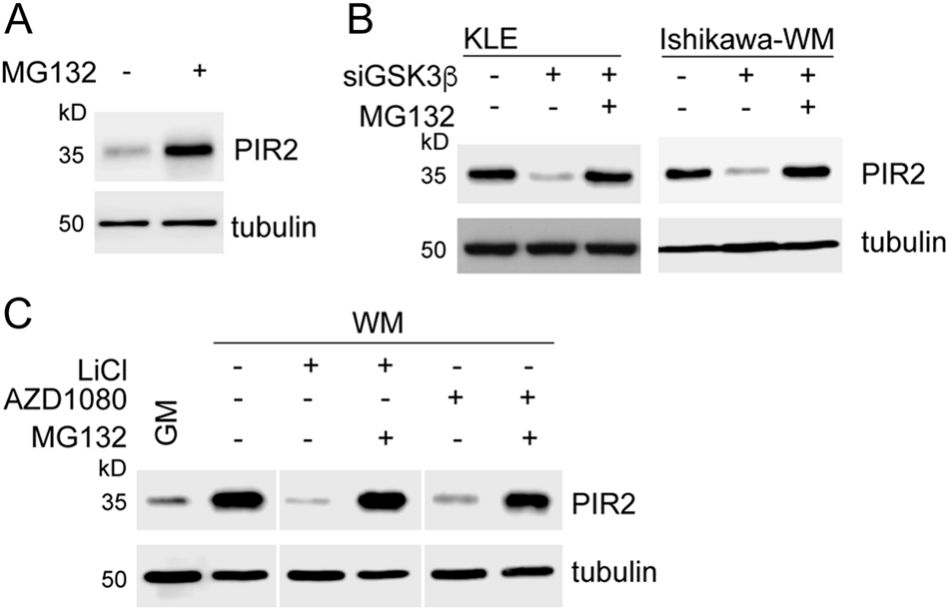
Phosphorylation of PIR2 downstream of GSK3β protects it from proteasomal degradation. **a** Western blot showing accumulation of PIR2 in Ishikawa cells in growth medium (GM) following inhibition of proteasome activity by MG132 treatment. **b** GSK3β was depleted in KLE cells and Ishikawa cells in WM, and treated with 25 uM MG132 for 4 h before collection. Lysates were used for western blotting. **c** Ishikawa cells in WM for 4 days were treated with GSK3β inhibitors LiCl (25 mM) or AZD1080 (100 nM) for 24 h. Where indicated MG132 (25 uM) was added to culture media 4 h before collection of cells. PIR2 expression was assessed by western blotting. Tubulin was used as equal loading control in all western blots

Proteasomal degradation is among the major pathways that regulate protein turnover in cells^33,34^. As PIR2 expression is regulated predominantly at protein level in EC, we asked whether inhibition of proteasome activity would affect PIR2 levels. As a proof of principle experiment, we treated Ishikawa cells in GM with the proteasome inhibitor MG132 and analysed the changes in PIR2 protein levels (Fig. 4a). Upon inhibition of proteasome activity, PIR2 levels increased, suggesting that in these cells PIR2 is constitutively degraded by the proteasome. To further assess the impact of GSK3β on PIR2 stability, we analysed PIR2 protein levels in GSK3β-depleted KLE cells and oestrogen-starved Ishikawa cells, in the presence or absence of the proteasome inhibitor MG132 (Fig. 4b). In both experimental settings, inhibition of proteasome restored PIR2 protein levels following GSK3β-depletion, demonstrating that phosphorylation of PIR2 downstream of GSK3β protects it from proteasomal degradation (Fig. 4b).

To further confirm the role of GSK3β in the inhibition of PIR2 degradation, we treated oestrogen-starved Ishikawa cells with GSK3β inhibitors lithium chloride and AZD1080 (Fig. 4c). While both inhibitors induced loss of PIR2 protein, treatment of cells with MG132 restored PIR2 levels. Treatment of KLE cells with GSK3β inhibitors also confirmed these findings (Supplementary Figure S4).

### Inhibition of PIR2 expression by GSK3β inhibitors impairs EC cell proliferation

**Fig. 5:**
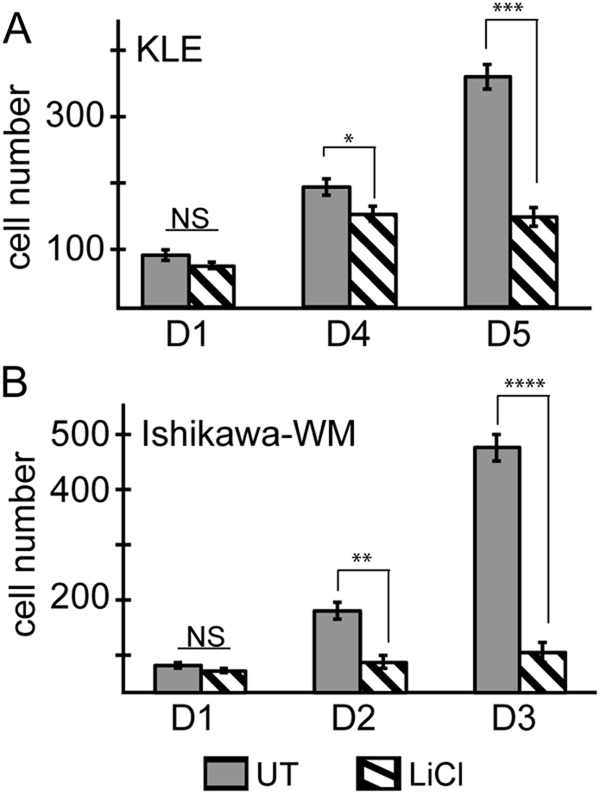
LiCl inhibits EC cell proliferation. KLE cells (**a**) and Ishikawa cells in WM (**b**) were treated with 25 mM LiCl and proliferation was assessed following fixing cells on indicated days. Graphs were generated following equalisation of the number of initially plated cells to 100. (NS: not significant, *P value < 0.05, **P value < 0.01, ***P value < 0.001, ****P value < 0.0001calculated by Student’s *t* test. Error bars represent standard deviations of experiment replicates)

To assess the impact of modulation of PIR2 stability by GSK3β inhibitors on cell proliferation, we first treated KLE cells with LiCl and analysed cell growth dynamics for up to 5 days. As shown in Fig. 5a, LiCl impaired proliferation significantly, supporting previously published effects of GSK3β inhibitors on EC cell proliferation^24^. We also tested the effect of LiCl on oestrogen-starved Ishikawa cells (Fig. 5b). While untreated cells proliferated rapidly, LiCl treatment had a severe cytostatic effect on these cells. The effect of LiCl on cell proliferation was more prominent compared to PIR2 depletion, potentially due to effect of lithium on multiple cellular signalling pathways. Nevertheless, our data suggest that expression of PIR2, downstream of GSK3β, is an important mechanism that drives EC cell proliferation and inhibition of the GSK3β-PIR2 signalling axis impairs proliferation in EC.

### GSK3β-PIR2 axis in endometrial tumours

**Fig. 6:**
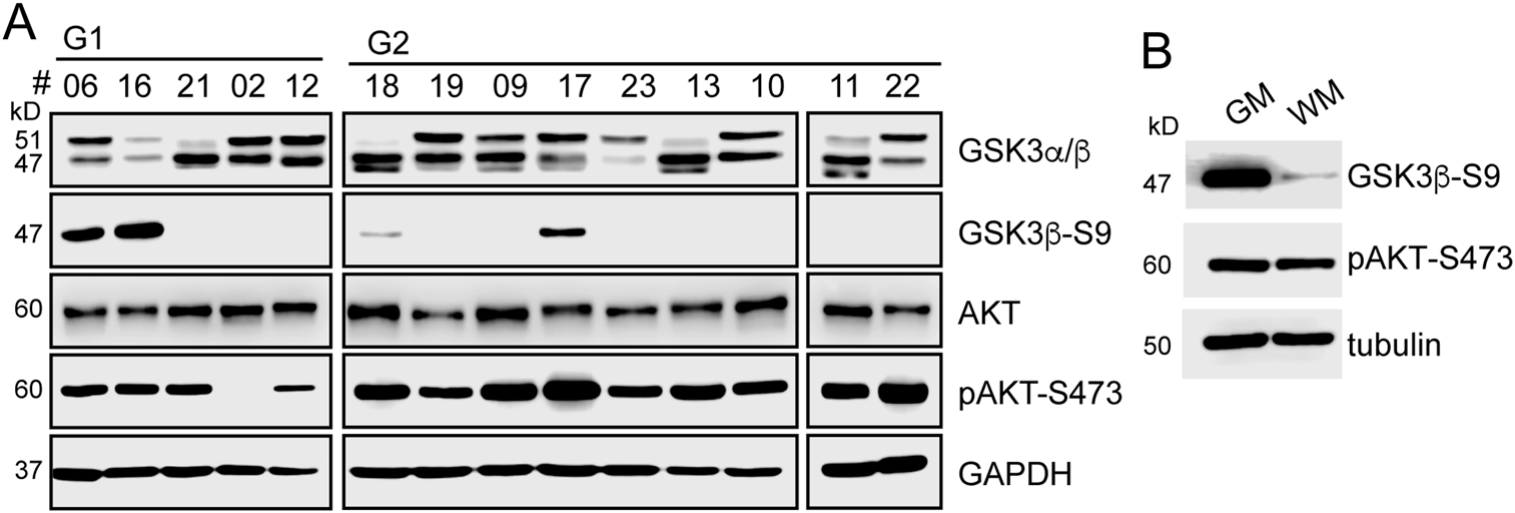
AKT-GSK3β axis in EC. **a** Tumour samples used in Fig. 1 were used to analyse the expression and activity of GSK3β and AKT in EC by western blotting. **b** S9-phosphorylation of GSK3β in relation to AKT activity was assessed in Ishikawa cells in GM and WM. Tubulin was used to assess equal protein loading

After characterising the GSK3β-PIR2 signalling axis in EC cell lines, we sought to investigate whether a similar axis was also present in tumour samples. Analysis of GSK3β and S9-GSK3β levels in our tumour panel demonstrated that GSK3β is in active state in the majority of the tumours (Fig. 6a). These data show that in EC, GSK3β is important for maintenance of tumorigenic phenotype and that there is a correlation between GSK3β activity and PIR2 expression *in vivo* (Fig. 6a).

In normal endometrium and in breast cancer cell lines, GSK3β is typically inactivated by the oestrogen receptor-AKT pathway^21,35–37^. If GSK3β were indeed critical for EC tumour maintenance, then it would be plausible to hypothesise that AKT-GSK3β axis would be impaired in endometrial tumours, to maintain high levels of GSK3β activity. To test this hypothesis, we analysed the tumour samples for the active form of AKT. It has been reported that >80% of EC tumours harbour mutations that target and activate AKT^17–20^ and in parallel with this, we detected S473-AKT in 91% of the samples (Fig. 6a). However, we did not detect a correlation between activation of AKT and inactivation of GSK3β, suggesting that in EC, AKT-GSK3β signalling axis is not functional.

To provide further support to this hypothesis, we assessed the AKT-GSK3β axis by comparing S473-AKT levels in the Ishikawa cells in GM and WM. As reported previously, these cells express high levels of inactive GSK3β and active AKT, under normal growth conditions (GM)^38^. However, in response to oestrogen withdrawal, although S9-GSK3β levels were reduced dramatically, we could not detect a significant change in S473-AKT levels (Fig. 6b). These data support our finding in tumour samples demonstrating that GSK3β is not regulated downstream of AKT.

## Discussion

Although most women with EC are cured by surgery and radiotherapy, nearly 20% present with aggressive disease that has an extremely poor prognosis. Improving outcomes for these patients requires early diagnosis and effective targeted therapy, which in turn necessitates the identification of endometrial cancer biomarkers^39,40^.

Several oncogenes and tumour suppressor genes have previously been identified as biomarkers in EC, including K-ras^41^, HER2^42^, PI3KCA^43^, FGFR2^44^, PTEN^45^ and p53^46^. While most of these serve as prognostic predictors, only a few stand out as potential therapeutic targets^47^. Here we have shown, by using a panel of normal endometrial tissue, endometrial cancer samples and EC cell lines that PIR2 (i) is exclusively expressed in EC and not in normal endometrium (Fig. 1), (ii) is important for EC cell proliferation (Fig. 1), and (iii) can be targeted by using GSK3β inhibitors (Figs. 3 and 4); substantiating PIR2 is a potential targetable biomarker for EC.

As stated by Hanahan and Weinberg, “*sustaining proliferative signalling is the most fundamental trait of cancer cells*”^48^. We have previously shown that PIR2 is critical for the proliferation of keratinocytes and HNSCC cells^29^ and here, we showed that PIR2 is also important for the proliferation in EC cells, especially in the absence of steroid hormone receptor mediated mitogenic signals (Fig. 1). Of note, PIR2 expression was detected in 14 out of 14 tumour samples (100%). Taken together, these finding suggest that PIR2 functions as an oncogene.

To assess the function of PIR2 in EC and to investigate the molecular mechanisms that regulate its cellular levels, we used two EC cells lines as model systems. Ishikawa cells, which express relatively lower levels of PIR2, are ER-positive and also express high levels of S473-AKT. Here, depletion of PIR2 expression did not cause a significant cytostatic effect, potentially due to the powerful effects of ER and AKT on cell proliferation. Indeed, when we inhibited ER signalling, we observed increased PIR2 expression, which was associated with proliferation. Here, the cells which failed to increase PIR2 expression, showed signs of impaired proliferation (Fig. 1f). KLE cells, on the other hand, are ER-negative^49^ and express low levels of S473-AKT^38^, and as such are in need for alternative oncogenic signals. This may explain why this cell line expresses higher levels of PIR2 protein.

A thorough investigation revealed that although PIR2 transcript is ubiquitously expressed in the endometrium and in EC, its protein levels are tightly regulated downstream of GSK3β, where un-phosphorylated PIR2 protein is degraded by the proteasome (Fig. 3, Fig. 4). In normal endometrium, GSK3β activity changes throughout the menstrual cycle: up to 5-fold higher levels of inactive GSK3β during secretory phase (low oestrogen, high progesterone), compared to the proliferative phase (high oestrogen, low progesterone)^50^. EC typically targets post-menopausal women with excessive un-opposed oestrogen activity (high oestrogen, low progesterone), as such creates an environment very similar to the proliferative phase of the menstrual cycle, with high levels of active GSK3β. This also supports our data on the lack of S9-GSK3β in the tested tumour samples (Fig. 6).

GSK3β serine/threonine kinase has been implicated in many diseases and disorders including metabolic, immune and neurological disorders, aging and cancer^26^. Its role in cancer is complex and controversial. While it is overexpressed and drives proliferation in glioblastoma, pancreatic cancer, ovarian cancer, prostate cancer, multiple myeloma, acute lymphocytic leukaemia, acute myeloid leukaemia and chronic lymphocytic leukaemia, it functions as a tumour suppressor in breast cancer and medullablastoma^26,51–54^. In endometrial cancer cell lines, GSK3β inhibitors exhibit anti-proliferative effects^24,25^. This effect was shown in both ER-positive and ER-negative EC cell lines. Our results, however, demonstrate that in ER-positive cells in the presence of oestrogen, GSK3β is predominantly inactive, as evidenced by high levels of S9-GSK3β isoform (Figs. 3d and 6b). This inhibitory effect of oestrogen on GSK3β has previously been shown in brain, in particular, in association with tau phosphorylation^55,56^. Therefore, as GSK3β in ER-positive EC cell lines is already inactive, one explanation for the cytostatic effect of GSK3β inhibitors in ER-positive EC cell lines could be due to the off-target actions of the kinase inhibitors used in these studies.

In order to advance the outcomes of cancer treatments, it is of vital importance to develop molecularly targeted therapeutics. Although use of GSK3β inhibitors, based on their *in vitro* and *in vivo* cytostatic effects, might sound like a promising approach to treat EC, their effect on normal endometrium should also be considered thoroughly. Our findings, however, show that PIR2 is not expressed in normal endometrium, suggesting that potential specific PIR2 inhibitors could be used for the treatment of EC, without the risk of harming the normal uterine. This cancer specific expression profile of PIR2 and its targetable nature presents an exciting opportunity to develop therapeutic agents to treat EC.

## Materials and methods

### Cell culture

All cell lines were grown as recommended by ATCC. For withdrawal experiments, Ishikawa cells were washed at least three times with PBS and were cultured in phenol red free DMEM containing 10% Charcoal Stripped FBS. Experiments in withdrawal medium (WM) were performed following growth of Ishikawa cells in WM for 4–5 days to erase ER activation memory.

### Treatments and transfections

Lipofectamine-2000 or RNAiMAX (Invitrogen) was used for transfections as described previously^57^. Predesigned siRNA targeting PIR2, and GSK3β were purchased from Ambion Ltd. MG132 was from Bio-Mol, LiCl was from Sigma and AZD1080 was from Selleckchem.

### Plasmids and stability assay

Human PIR2 cloned in pcDNA3.1 without any tags was used for stability assays. QuikChange II Site-Directed Mutagenesis Kit was used to generate the phosphomimetic mutants as described previously^58^. Stability assays were performed following transfection of H1299 cells with indicated plasmids. Briefly, 6 h after transfection, cells were split into 6 cm plates and 24 h later treated with 75 μm CHX. Cells were collected at indicated time points and cell lysates were run on acrylamide gels. PIR2-antibody^27^ reactive bands were quantified by using ImageJ software and normalised results were plotted on a chart.

### Mass spectrometry and western blot analysis

For mass spectrometry, PIR2 was overexpressed in HEK293 cells and immunoprecipitated by using anti-PIR2 antibody. 24 h following transfection, cells were collected and lysed in triton buffer [50 mM Tris-HCl (pH 8.0), 150 mM NaCl, 1 mM EGTA (pH 8.0), 100 mM NaF (pH 8.0), 10% glycerol, 1 mM MgCl2, 1 mM sodium orthovanadate, 1% (v/v) Triton X-100 and complete protease inhibitors (Roche)]. PIR2 protein was precipitated by incubating the cell lysates with the PIR2 antibody. Immunoprecipitates were run on an acrylamide gel and the bands were revealed by colloidal Coomassie stain. Following de-staining, a fraction of PIR2 band was removed from gel and subjected to mass spectrometry following incubation with proteolytic enzymes. For western blot analysis, tissues were homogenised and cell pellets were sonicated in lysis buffer (25 mM Tris (pH.6.8), 7% glycerol, 0.8% SDS). Proteins were denatured, separated on SDS polyacrylamide gels, and then transferred to nitrocellulose membranes. The following antibodies were used at dilutions suggested by the supplier: PIR2 antibody was generated as described before^27^. Actin (sc1616), tubulin (sc-8035), GAPDH (sc-32233), GSK3α/β (sc-7291) were from Santa Cruz. S9-GSK3β (#5558), AKT (#4691) and S473-AKT (#4060) antibodies were from Cell Signalling. Immunoreactive proteins were detected using an enhanced chemiluminescence system (Thermo Fisher Scientific Inc).

### Polymerase chain reaction

RNA extraction and cDNA synthesis were done as described before^59^. Equal amount of RNA was used in cDNA synthesis. Quantitative PCRs were performed with TaqMan Universal Master mix with MJ Research DNA Engine Opticon2 or with QuantStudio-3 Real-Time PCR system. All TaqMan assays were purchased from Applied Biosystems. For semi-quantitative PCR the quality of cDNA was tested by GAPDH amplification (GF: 5′-GGCTGAGAACGGGAAGCTTGTCAT-3′ and GR: 5′-CAGCCTTCTCCATGGTGGTGAAGA-3′). PIR2 expression was analysed with the primers: FL-PIR2-F: 5′-ATGGGCTCAGCTGGTAGGC-3′ and FL-PIR2-R: 5′-GGTTGTGGATGGGTCGTGCT-3′. The amplified DNA fragments were analysed as described previously^60^.

### The Cancer Genome Atlas (TCGA) RNA-seq data analysis

RNA-seq data from normal (*n* = 24) and tumour (*n* = 176) samples were downloaded from the TCGA-Uterine Corpus Endometrial Carcinoma project. The normalised RSEM value of PIR2 was extracted and compared between the tumour cohort and normal cohort. Statistical significance was calculated by the Mann–Whitney–Wilcoxon test in R.

### Proliferation assay

For proliferation assays, cells were seeded in triplicate/quadruplicate in 24-well plates and fixed in 50/50% acetone/methanol at indicated times. Following staining with DAPI, images were acquired by using UV filter. The stained nuclei were counted using ImageJ software.

### Cell cycle and apoptosis assays

For cell cycle and apoptosis assays, cells were collected, washed in PBS and re-suspended in hypotonic fluorochrome solution, made by diluting 1 mg/ml stock PI solution 1:20 (Sigma, UK) in hypotonic buffer (0.1% (w/v) sodium citrate, 0.1% (v/v) Triton X-100). The suspension was analysed by using BD Accuri™ C6 flow cytometry (BD biosciences, UK).

### Immunohistochemistry

All tissue samples were taken from the BRC BioBank (Biobank ethics ref.: ^14^/NW/1260, HTA licence number: 12552). Paraffin sections were deparaffinized and stained with anti-PIR2 by using the DAKO DAB + Substrate Chromogen System (DAKO-K3467).

### Screening of PKIS

N-TERT cells were treated with 100 nM of each drug and collected 5 h later. PIR2 levels were then assessed by western blotting and compared to that of untreated cells.

## Electronic supplementary material

Supplementary Figure Legends

Supplementary Table Legend

Summary of the data showing the list of compounds from the GSK-PKIS that induced PIR2 protein loss

PIR2 up-regulation is essential for adaptation to oestrogen free milieu

PIR2 transcript levels in EC cell lines, in normal endometrium and EC samples

PIR2 is a phosphoprotein

Inhibition of proteasome activity restores PIR2 protein levels
