# Supplementary material for: Pir2/Rnf144b is a potential endometrial cancer biomarker that promotes cell proliferation
Source: Cell Death Dis. 2018 May 2;9(5):504. doi: 10.1038/s41419-018-0521-1 (PMC5938710; doi:10.1038/s41419-018-0521-1)
Supplement: Supplementary file 1 — Supplementary Figure Legends [file 41419_2018_521_MOESM1_ESM.docx]

**Supplementary Figure Legends**

**Supplementary Figure S1: PIR2 up-regulation is essential for adaptation to oestrogen free milieu**. **(a)** Loss of PIR2 affects cell cycle profile. PIR2 was depleted in KLE cells. Sub-G1 population and cell cycle profile was analysed by flow cytometry on indicated days. (NS: not significant, *P-value <0.05, **P-value <0.01, calculated by Student’s t-test. Error bars represent standard deviations of experiment replicates.)

**(b)** Ishikawa and Hec1A cells in growth media were transfected with scrambled control siRNA (scr) or siRNA targeting PIR2 (siPIR2). Cells, plated in triplicates, were fixed on indicated days and counted following DAPI staining. Error bars represent standard deviations of experiment replicates (n=3). Western blot shows PIR2 levels on the fifth day of the experiment. **(c)** Proliferation assay showing effect of oestrogen withdrawal on Ishikawa cell proliferation. **(d)** Schematic representation of PIR2 expression in Ishikawa cells in GM or WM, following transfection with scrambled control or siPIR2. Cell lysate from Ishikawa cells in GM and in WM for 1, 3 and 5 days were run on acrylamide gels. PIR2 expression was assessed by western blotting. Band intensities were plotted on the graph following quantification by the ImageJ software.

**Supplementary Figure S2. PIR2 transcript levels in EC cell lines, in normal endometrium and EC samples.** PIR2 transcript levels in EC cell lines **(a)**, Ishikawa cells growing in GM or in WM **(b)**, normal endometrium **(c)**, and tumour samples **(d)**, were assessed by semi-quantitative (SQ) RT-PCR. GAPDH was used to assess equal RNA input.

**Supplementary Figure S3: PIR2 is a phosphoprotein.** S38, S250 and S301 residues were changed into aspartate residues, by site-directed mutagenesis (S38D, S250D and S301D). Wild type PIR2 and phosphomimetic mutants were overexpressed in H1299 cells. Cells were treated with cycloheximide and collected at 6H, 12H and 24H time points. Cell lysates were probed for PIR2. Tubulin was used as equal loading control.

**Supplementary Figure S4: Inhibition of proteasome activity restores PIR2 protein levels.** KLE cells were treated with GSK3β inhibitors LiCl (25 mM) or AZD1080 (100 nM) for 24 hours. Where indicated MG132 (25 uM) was added to culture media 4 hours before collection of cells. PIR2 expression was assessed by western blotting. Tubulin was used as equal loading control.
