# Supplementary material for: Pir2/Rnf144b is a potential endometrial cancer biomarker that promotes cell proliferation
Source: Cell Death Dis. 2018 May 2;9(5):504. doi: 10.1038/s41419-018-0521-1 (PMC5938710; doi:10.1038/s41419-018-0521-1)
Supplement: Supplementary file 2 — Supplementary Table Legend [file 41419_2018_521_MOESM2_ESM.docx]

**Supplementary Table Legend**

**Supplementary Table S1: Summary of the data showing the list of compounds from the GSK-PKIS that induced PIR2 protein loss.** N-TERT cells treated with each of the 341 compounds and cell lysates were analysed by western blotting. The most effective compounds were chosen on the basis of an 85% cut-off PIR2-downregulation.
