# Supplementary figures and images for: Pir2/Rnf144b is a potential endometrial cancer biomarker that promotes cell proliferation
Source: Cell Death Dis. 2018 May 2;9(5):504. doi: 10.1038/s41419-018-0521-1 (PMC5938710; doi:10.1038/s41419-018-0521-1)

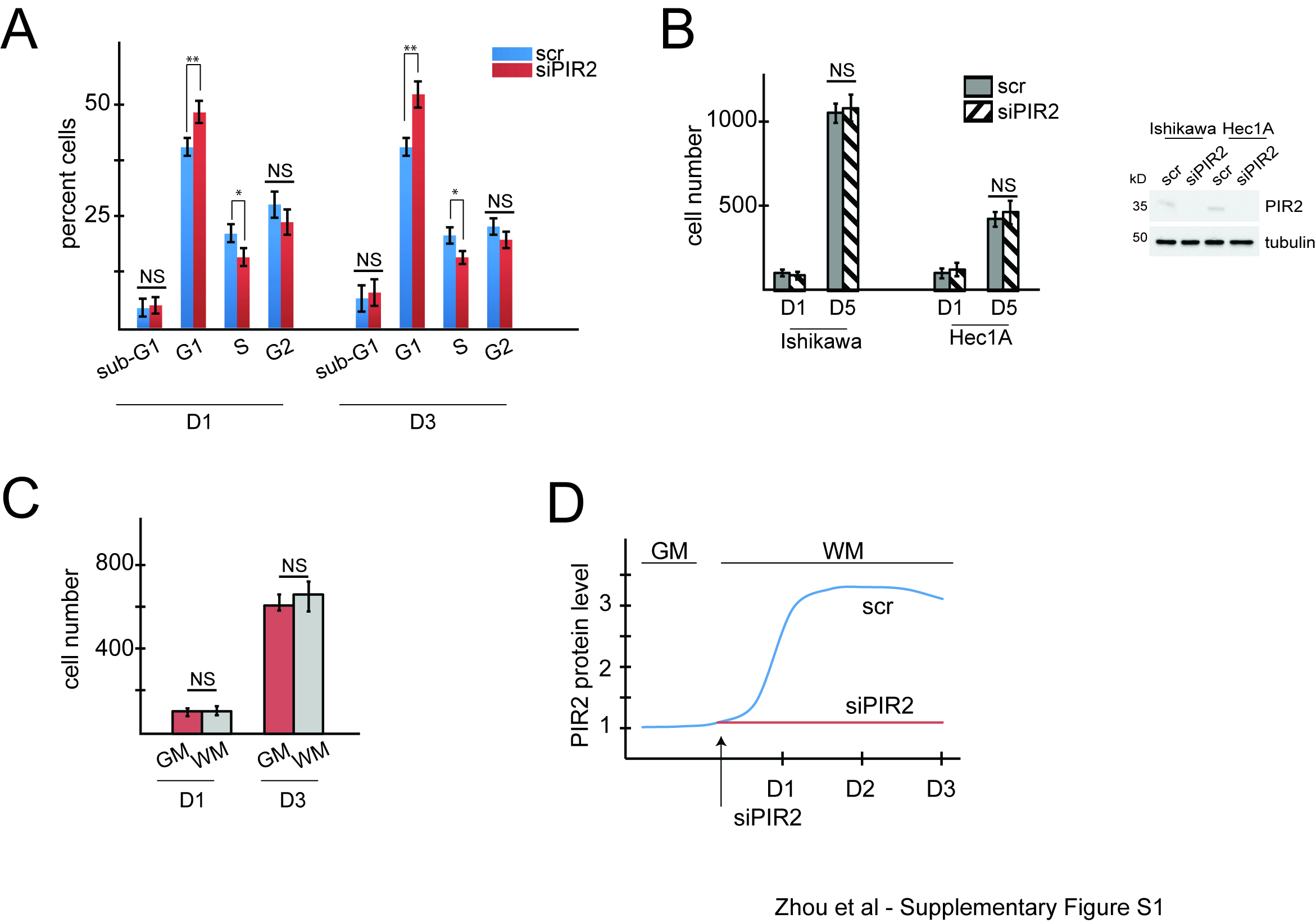

Supplement: Supplementary file 4 — PIR2 up-regulation is essential for adaptation to oestrogen free milieu [file 41419_2018_521_MOESM4_ESM.tif]

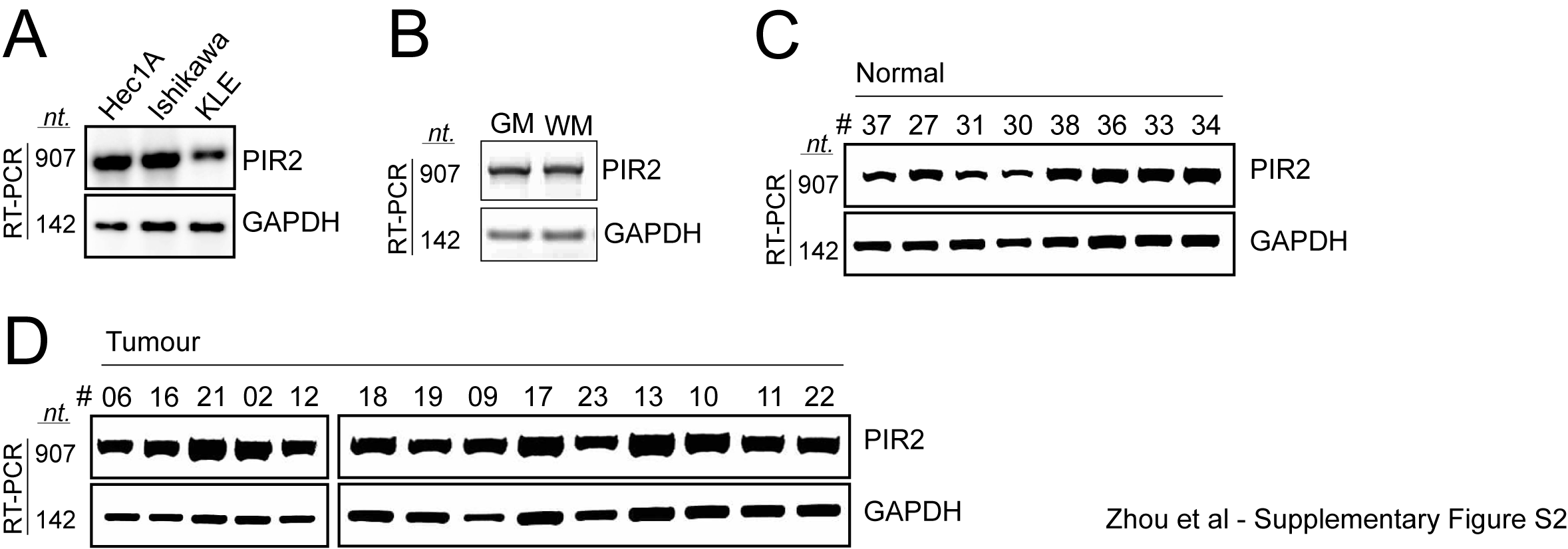

Supplement: Supplementary file 5 — PIR2 transcript levels in EC cell lines, in normal endometrium and EC samples [file 41419_2018_521_MOESM5_ESM.tif]

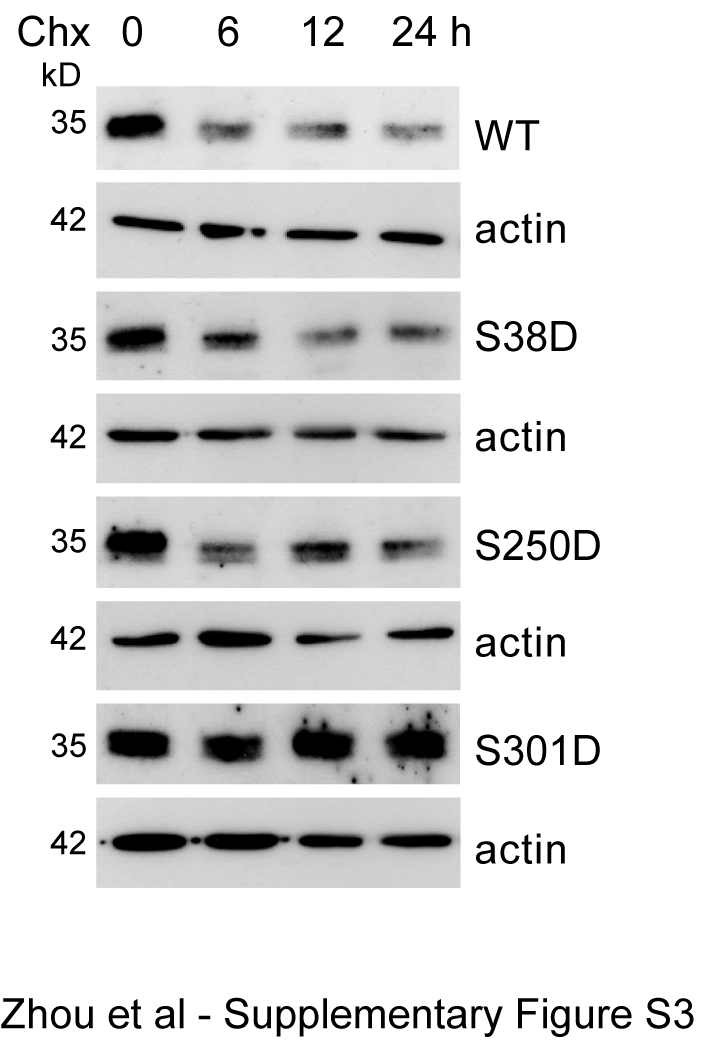

Supplement: Supplementary file 6 — PIR2 is a phosphoprotein [file 41419_2018_521_MOESM6_ESM.tif]

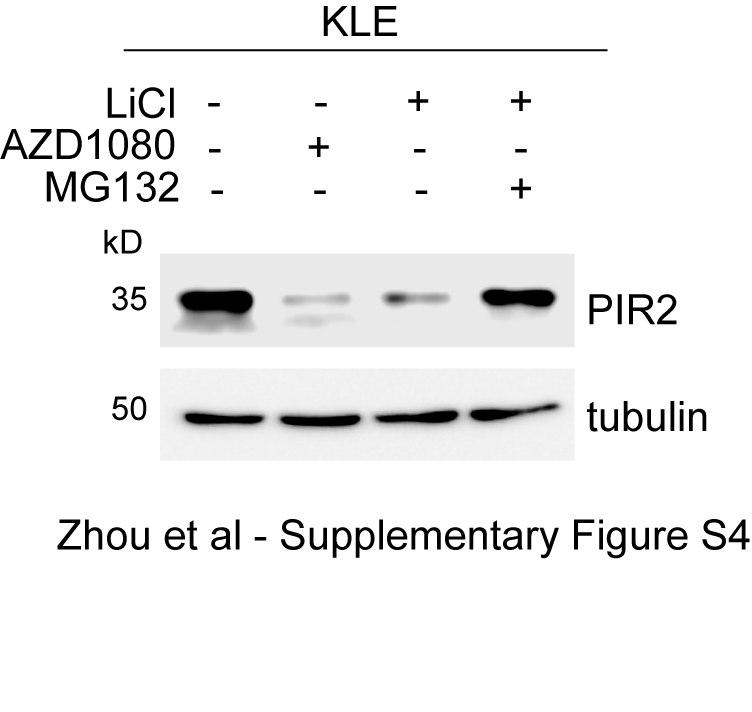

Supplement: Supplementary file 7 — Inhibition of proteasome activity restores PIR2 protein levels [file 41419_2018_521_MOESM7_ESM.tif]
